# A Mixed Methods Study of Medication Adherence in Adults with Neurofibromatosis Type 1 (NF1) on a Clinical Trial of Selumetinib

**DOI:** 10.3390/cancers17020295

**Published:** 2025-01-17

**Authors:** Millicent S. Curlee, Mary Anne Toledo-Tamula, Melissa Baker, Daniel Wikstrom, Cynthia Harrison, Amanda Rhodes, Margaret Fagan, Cecilia Tibery, Pamela L. Wolters, Brigitte C. Widemann, Andrea M. Gross, Staci Martin

**Affiliations:** 1 Pediatric Oncology Branch, Center for Cancer Research, National Cancer Institute, 9030 Old Georgetown Road, Bethesda, MD 20892-8200, USA; milly.curlee@nih.gov (M.S.C.); maryanne.tamula@nih.gov (M.A.T.-T.); mb4341@mynsu.nova.edu (M.B.); dan.wikstrom@nih.gov (D.W.); cynthia.harrison@nyulangone.org (C.H.); margaret.fagan@nih.gov (M.F.); woltersp@mail.nih.gov (P.L.W.); widemanb@mail.nih.gov (B.C.W.); andrea.gross@nih.gov (A.M.G.); 2 Clinical Research Directorate, Frederick National Laboratory for Cancer Research, Leidos Biomedical Research, Inc., Frederick, MD 21702, USA; cecilia.tibery@nih.gov

**Keywords:** neurofibromatosis, plexiform neurofibroma, selumetinib, medication adherence, qualitative interview, medication event monitoring system, pill count, medication diary, clinical trial

## Abstract

Background: Oral therapeutic options for plexiform neurofibromas (PNs) in individuals with neurofibromatosis type 1 (NF1) are receiving attention in clinical research. The MEK inhibitor (MEKi) Selumetinib is FDA-approved in children ages 2+ years with inoperable PNs, and shows activity in adults. Prolonged therapy with selumetinib is necessary to maintain tumor reduction. Therefore, investigating long-term adherence is vital to understand patterns of adherence over time and its impact on clinical outcomes. Mixed methods research offers rich information about adherence that can inform future intervention trials, and can assist practitioners in addressing medication adherence concerns. Methods: This mixed-method pilot study is the first examination of the feasibility of a technology-based adherence assessment method, the medication events monitoring system (MEMS^TM^), among individuals with NF1-PN. Adherence was monitored in a small sample of patients (N = 12; mean age = 34.36 years; 58% male) with NF1 and PN across eighteen 28-day treatment cycles. Qualitative data were obtained from individual interviews using inductive and deductive techniques for thematic analysis. Results: The predetermined criterion was met, suggesting that using MEMS^TM^ is feasible despite some challenges with the caps. Depression and overall stress were significantly related to reduced adherence, although these results should be considered hypothesis-generating. Barriers to medication adherence included forgetting and the timing of doses related to eating. Facilitators included consistency, reminders, and social support. Conclusions: This study highlights patient characteristics that may be related to increased risk for nonadherence, as well as challenges with electronic pill caps that should be considered in future clinical trials for NF1-related PN. Results can inform future adherence interventions for adults with NF1 and PNs. Future research with larger samples is needed to fully explore factors related to long-term medication adherence among individuals with NF1.

## Simple Summary

People with neurofibromatosis type 1 (NF1) may develop tumors called plexiform neurofibromas (PNs). Selumetinib was the first oral medication to gain FDA approval to treat PNs in children, and the drug also has activity in adults. Clinical observations suggest that people must continue taking selumetinib to maintain its effects. Therefore, it is important to research how well people take selumetinib as prescribed over a long period of time. We used electronic pill caps that record when the bottle is opened, pill counts, and self-report diaries to measure adherence over eighteen 28-day treatment cycles. We found that using the caps is feasible but presents some challenges. We also found evidence that depression and stress were related to lower adherence in our small sample. We also interviewed patients, who talked about things that make adherence easier (consistency, reminders, and social support) and more difficult (forgetting and dose timing).

## 1. Introduction

Neurofibromatosis Type 1 (NF1) is an autosomal dominant disorder that affects approximately 1 in 2132–4712 people [1] and can lead to the development of histologically benign peripheral nerve sheath tumors, known as plexiform neurofibromas (PNs). PNs occur in around 25–60% of people with NF1 (NF1-PN) and are most likely congenital [2]. Internal PNs often are not diagnosed until they are large and cause clinically relevant symptoms [3]. PNs are related to both morbidity and mortality in patients with NF1, as they can cause a wide variety of symptoms depending on the size and location of the tumor [4]. Other PN complications include airway obstruction, visual deficits, pain, and nerve function impairment [5,6,7,8]. Natural history studies show that the presence of PNs may impact psychosocial functioning and quality of life [9,10].

Until recently, the most common method for addressing PNs was resection; however, surgery is often contraindicated because tumors can extend into vital tissues [2,11] and can re-grow following partial resection [5,6,12]. To address this issue, clinical trials have focused increasingly on oral therapeutic options [13]. The first pediatric trial of the oral MEK 1/2 inhibitor selumetinib (the SPRINT trial; AZD6244) found that selumetinib resulted in PN reduction and clinical benefits in children [12], leading to the only FDA drug approval for children 2+ years with inoperable tumors [5,6,12]. An adult selumetinib trial also documented reduced tumor size and decreased tumor pain intensity and pain interference [14]. Medication adherence is crucial in clinical trials, where nonadherence can interfere with efficacy.

Although clinical trial participants tend to have greater medication adherence than the general population [15], poor adherence may under-power analyses and lead to incorrect conclusions [16,17]. In the SPRINT trial, adherence during the first three 28-day cycles was excellent per caregiver-completed diaries (99%) and staff-completed pill counts (98%) [13]. However, it has yet to be determined whether the dosing schedule or possible side effects (e.g., nausea, acneiform rash) may inhibit consistent medication-taking. In the phase 2 trial investigating selumetinib in adults, participants were initially prescribed 75 mg of selumetinib twice a day, approximately 12 h apart, for a 28-day cycle [14]. However, the study was amended following the first two participants’ experience of dose-limiting skin toxicity. All other participants received a starting dose of 50 mg twice a day [14]. The medication was available in 10 mg or 25 mg capsules, so participants had to take several capsules for each dose. As complicated treatment regimens have been associated with worse adherence in other populations [18], it is necessary to investigate patients’ adherence in a trial that requires both the specific timing of doses and food restriction. During the clinical trial, abstinence from eating two hours before and one hour after dosing was required, which may pose adherence challenges. Following the completion of this study, recent research led to changes in the FDA label [19]; selumetinib can now be taken with or without food [20].

Additionally, early clinical data suggested that prolonged treatment may be necessary to maintain efficacy [13]; therefore, research on long-term medication adherence is vital for assessing the impact on medical outcomes and implementing behavioral interventions targeting factors that put patients at risk for non-adherence. Studies examining other populations have found decreased medication adherence over time [21,22,23,24], suggesting the need to examine longitudinal adherence patterns in individuals with NF1-PN.

Compared to self-report and pill counts, a technology-based adherence assessment method is the medication events monitoring system (MEMS^TM^), in which pill caps record dates and times of bottle openings [25]. MEMS^TM^ caps are useful for assessing adherence to medications with specific timing requirements (e.g., selumetinib). Some past research has shown electronic caps to be more reliable compared to self-report diaries [26,27], and have even been used to identify over-adherence (e.g., people who take more pills than prescribed) [28]. Further, the caps may be particularly suited to clinical trials, where reliable adherence data can help investigators have confidence in their findings related to efficacy. Thus, they may offer advantages over more traditional methods of adherence measurement. Challenges include changing medication-taking routines, transferring medication to the bottle fitted for the cap [29], and cap malfunctioning [26]. To date, no studies have assessed the feasibility of MEMS^TM^ caps for the NF1-PN population.

### Study Rationale and Aims

The primary objective of this mixed method pilot study was to assess the feasibility of using MEMS^TM^ caps to monitor medication adherence in adults with NF1-PN on a clinical trial. Given the small sample size, all other objectives were considered exploratory. These include: (1) to document longitudinal patterns of adherence; (2) to identify patient characteristics and/or time periods during treatment that are related to poor adherence; (3) to compare adherence between MEMS^TM^ caps, pill counts, and diaries. The exploratory objective of the qualitative methods was to explore barriers and facilitators to adherence through semi-structured interviews with adults with NF1 on a clinical trial of selumetinib. Even with small sample sizes, qualitative data are vitally important to identify barriers and facilitators for adherence and can help inform adherence-promoting interventions for individuals enrolled in clinical trials.

## 2. Methods

### 2.1. Eligibility

Eligibility criteria included a confirmed diagnosis of NF1, aged 3–59 years, ability to understand and sign a written informed consent, ability to read and comprehend English, regular access to an electronic device with internet access, and enrollment on a clinical trial for selumetinib (manufactured by AstraZeneca/Alexion, Cambridge, UK) concomitantly with their participation in this adherence study. The clinical trial required participants to take selumetinib twice a day, twelve hours apart, and to abstain from eating for two hours before and one hour after the dose.

### 2.2. Measures

#### Baseline

Background Information. Participants provided information about demographic characteristics and medical status, and characterized their NF symptoms and their pain (separately) as mild, moderate, or severe.

Life Events Checklist (LEC). Participants completed the Life Events Checklist, adapted from Elliott-DeSorbo et al. [30], to document stressful events in the past three months. The score is the number of stressful events endorsed (range 0–22).

Rating of Overall Stress Scale (ROSS). Participants rated their stress in the past month from 0 (not stressed) to 10 (very stressed). This measure was adapted from Leidy et al. [31].

Quality of Life. The following PROMIS self-report short forms were administered: depression (8 items) [32], pain interference (6 items) [33], and cognitive function (8 items) [34]. Raw scores were converted to T-scores (M = 50, SD = 10). Subscales had excellent internal reliability (α = 0.916–0.972).

### 2.3. Adherence

Pill Counts. At re-staging visits, a study team member documented the number of returned capsules, drug holds, and dose changes. Adherence was calculated by dividing the doses correctly taken by the number prescribed. During drug holds, doses were considered adherent if they were *not* taken. Twice, a pill bottle was lost by participants, so the pill count was missing.

Medication Diary. For each dose, participants were instructed to record the date, the time, and any comments. If a diary listed the time or number of capsules taken, researchers coded the dose as adherent. Empty cells were coded as non-adherent. One participant lost a diary for one cycle, but all other diaries were returned.

MEMS^TM^ Cap. Participants’ study drug bottles were fitted with a MEMS^TM^ cap at baseline. Having been given instructions to take the drug 12 h apart, adherence was defined as opening the bottle 11–13 h since their last dose. Data were considered missing if <50% of doses in the cycle were represented. A priori, the MEMS^TM^ feasibility target was ≥2 cycles monitored for ≥75% of participants. 

Barriers to Adherence Questionnaire. The barriers to adherence measure was created by the study team; participants were asked to think of times they missed a dose of their study drug in the past month and marked which of 16 possible reasons caused them to miss a dose. Participants also could provide additional reasons for missing medication.

Qualitative Adherence Interview. Participants completed a 15–30 min structured interview between cycles 8 and 12 (cycle = 28 days). Questions were about adherence barriers, facilitators, and advice participants would give to others.

### 2.4. Procedures

This study was approved by the National Institute of Health Intramural Institutional Review Board. Patients were approached and consented during their baseline visit on a selumetinib clinical trial [35] and provided informed consent for this adherence study (NCT03531814). For the clinical trial, participants completed 28-day treatment cycles and were instructed to take medication approximately 12 h apart. Routine protocol re-staging visits were performed before cycles 5, 13, and 19, and included the collection of adherence data. At each follow-up visit on the clinical trial, diaries and pill bottles (including empty ones) were collected, and MEMS^TM^ cap data were uploaded to Aardex MEMS^TM^ Adherence Software (ElectronReader 0.9.9). If adherence was <80% by any method, the team alerted the PI of the clinical trial, and adherence was discussed with the patient. Qualitative interviews were conducted by a licensed psychologist or trained psychology associate in a private clinic room (except for one that was over the telephone) and recorded for transcription. Participants completed the Barriers to Adherence questionnaire around the time of the interview.

### 2.5. Analyses

Quantitative. Quantitative analyses were conducted in SPSS 29.0.1. Pill count, diary, and MEMS^TM^ cap data were grouped across cycles 1–4, 5–8, 9–12, and 13–18. Adherence data were positively skewed, so non-parametric tests were used (i.e., Spearman correlations). Alpha was 0.05. As the primary outcome was feasibility, a power analysis was not conducted a priori. Given the small sample size, we were under-powered to perform some of the quantitative analysis. However, we report exploratory Spearman correlation results, as these require less power than analyses of variance comparing multiple groups (e.g., differences in adherence over time and differences between adherence assessment methods). Therefore, data comparing multiple groups are presented descriptively.

Qualitative. For qualitative data, thematic analysis was conducted using both deductive and inductive techniques. As the qualitative portion of this study was an exploratory aim, analyzing data for saturation was not part of the a priori methods. However, post hoc methods were conducted with thematic saturation defined as the point at which no new categories or codes were uncovered [36]. Retrospectively, the interview transcripts were reviewed in the order in which they were conducted to assess for new themes or subthemes. The initial coding dictionary was developed with broad themes that aligned with interview questions. The first two transcripts were coded by the last author, and subthemes were added to the dictionary as they arose. Next, themes and subthemes were discussed as a team (a licensed psychologist with extensive experience in qualitative research, a master’s level psychology associate, a postdoctoral fellow, and a post-baccalaureate fellow) while reviewing transcripts together, and modifications to the dictionary were made as needed to reach consensus. Subsequent transcripts were divided among the team and discussed collaboratively. All transcripts were reviewed by at least two team members.

## 3. Results

### 3.1. Participants

All 12 participants (M age = 34.36, SD = 8.91) who were approached agreed to enroll on this study (see Table 1 for demographics). Although the adherence study was open to ages 3+ years, the only treatment protocol open at our institution during data collection was for adults. All participants provided complete data with one exception: one participant was not given the Barriers to Adherence questionnaire due to researcher error. After the first 12 participants were enrolled on this protocol, the clinical trial of selumetinib closed to enrollment; thus, we were unable to enroll more participants on this adherence study.

### 3.2. Quantitative Findings

Feasibility of MEMS^TM^. Eleven of twelve participants (91.7%) completed at least the first two cycles; the 80% and 95% confidence intervals of the proportion are (75.8%, 98.1%) and (64.6%, 99.6%) based on Wilson’s method, respectively [37]. Due to battery expiration between restaging visits (n = 5), forgetting to replace the cap on the next bottle (n = 1), or forgetting to return the cap (n = 1), at least one cycle was unable to be collected for some participants. Additionally, one participant withdrew because they did not like using the MEMS^TM^ cap. One other participant’s cap malfunctioned, resulting in no data collected for that participant across any cycles. One participant was taken off the drug trial at cycle 9; therefore, their adherence data were only grouped for cycles 1–4 and 5–8.

**Figure 1:**
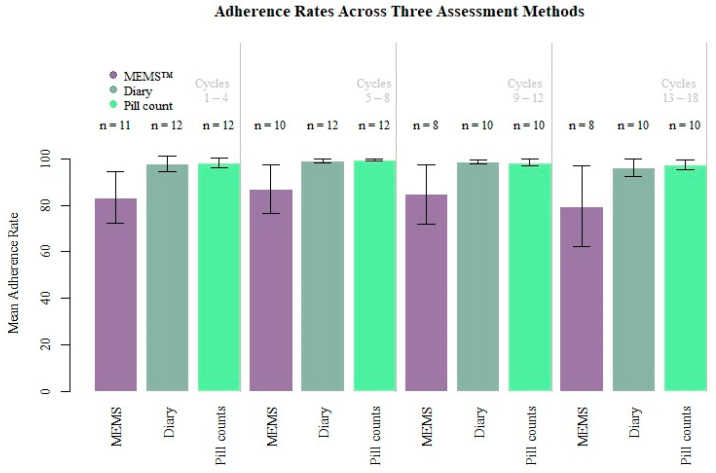
Adherence rates across three assessment methods.

Adherence Over Time. Adherence was above 90% over all time points, as measured by pill counts and diaries (Table 2; Figure 1). According to MEMS^TM^ caps, adherence remained relatively stable and ranged from 83.39% at cycles 1–5 to 79.68% during cycles 13–18. Pill count adherence rates ranged from 97.53% to 99.50%, while diary rates ranged from 96.14% to 99.22%.

Comparison of Adherence Assessment Methods. Given the lack of sufficient power to have confidence in results of statistical comparisons, these results are presented descriptively. As shown in Table 2, the mean MEMS^TM^ adherence rates over most cycles are lower than for diaries and pill counts, while the means for diaries and pill counts are fairly close to each other.

Patient Characteristics and Time Periods Related to Adherence Differences. Poorer MEMS^TM^ adherence rates were correlated with higher depression scores (Table 3). The better adherence from MEMS^TM^ and pill count data in cycles 1–4 is related to lower baseline overall stress. When examining differences in MEMS^TM^ adherence levels by baseline NF1 symptom severity and pain intensity, those with mild and moderate (vs. severe) symptoms and pain were grouped. Descriptively, there was not a clear pattern of differences in mean MEMS^TM^ adherence based on NF1 severity (Cycles 1–4: Mild/Moderate = 84.55, Severe = 82.01. Cycles 5–8: Mild/Moderate = 85.19, Severe = 89.73. Cycles 9–12: Mild/Moderate = 81.61, Severe = 90.03. Cycles 13–18: Mild/Moderate = 77.96, Severe = 82.54.). There was also no discernible pattern in differences in adherence based on pain intensity (Cycles 1–4: No pain/Mild/Moderate = 79.58, Severe = 90.07. Cycles 5–8: No pain/Mild/Moderate = 84.52, Severe = 90.74. Cycles 9–12: No pain/Mild/Moderate = 83.85, Severe = 87.50. Cycles 13–18: No pain/Mild/Moderate = 80.55, Severe = 77.08.).

### 3.3. Qualitative Findings

After coding all transcripts from the qualitative interviews, we reviewed them retrospectively to assess for saturation. The data reached saturation since no new subthemes were generated after the tenth participant.

### 3.4. Adherence Themes

A combined inductive and deductive thematic analysis resulted in subthemes spanning two major themes aligned with the interview questions: barriers to medication adherence (nine subthemes) and facilitators to medication adherence (10 subthemes).

### 3.5. Barriers to Adherence

#### Forgetting

Multiple patients described forgetting to take their study medication. One individual said, “Sometimes I forget”, and another replied, “Just forgetting”.

### 3.6. Memory/Cognitive Difficulties

Trouble remembering to take medication was a barrier distinct from momentary forgetfulness. Many individuals with NF1 have cognitive difficulties like attention deficits [38] and memory weakness [39]. One individual said “what has made it hard for me to take my meds is my memory. I have a really, really hard time remembering it. Um, like, ADHD, I guess”.

#### 3.6.1. Too Busy

One participant’s work schedule made it difficult to take their medication: “if I’m busy for work and I can’t eat at the same time that kind of messes up when I can take [the drug]”.

#### 3.6.2. Timing of Doses

The timing of doses made adherence challenging. Even participants who adhered well faced challenges complying with the drug’s required eating window (which has recently been removed from the FDA label [20]). One individual noted, “planning taking my medicine around social events, like if I’m going to go get dinner with friends or go out that night. Um, sort of timing it appropriately”. Another mentioned difficulty with timing medication during work, but followed this by sharing “It’s more like the issue also comes with the food aspect of the drug so… [the eating window] makes it really hard to plan activities. So sometimes I find myself having to, you know, take the drug two hours earlier than I planned”. Another participant described how social engagements can act as barriers, saying “I’m doing an activity with friends and I know I’m gonna eat. And I can’t just tell them, we can only do it at this time. So it’s more like life kinda gets in the way of the strict structure for the drug”.

#### 3.6.3. Being Away from Home

Not being home during dose times was a barrier, with one individual reporting, “The only time I’ve had a hard time is if I was out and I wasn’t home”. Another person shared, “if I’m on weekend and I try to go places and all, then I’ll… need to remember to take my medication with me [sic]”.

#### 3.6.4. Medical Symptoms

One participant’s stomach pain impacted their ability to follow the protocol’s eating requirements, saying, “figuring out my stomach is different, because I have a little bit of gastroparesis, so I have to wait a little bit, uh different time frames. So, figuring that out was a little bit challenging”.

#### 3.6.5. Keeping Pills in MEMS Bottle

For one participant, use of the MEMS caps was identified as a barrier if it conflicted with their preference for organizing medications. One participant described their concerns, “the pills were inside the bottle, my mind just didn’t associate that bottle with taking my meds because I didn’t see the pills so I would forget about it”. The patient further stated, “with the MEMS cap, I missed like two weeks’ worth of pills”. This patient withdrew from the adherence protocol early due to their difficulties with the MEMS cap.

#### 3.6.6. Sleep Schedule

Another barrier related to participants’ sleep schedules. One participant noted, “one time I overslept [past my dose time]”. Another elaborated, “sometimes it’s hard to stick to the exact times because, I mean, forcing yourself, in my case, to wake up at 4 in the morning—4 in the afternoon, it’s hard”.

#### 3.6.7. No Barriers

Two participants did not report any barriers; one participant replied, “What situations make it… I mean, there’s really no, no problem. I mean I take it as prescribed”.

### 3.7. Adherence Facilitators

#### 3.7.1. Consistency/Routine

A consistent schedule facilitated adherence. One individual said “Yeah, I have a schedule… basically I have a built-in alarm clock, basically. I’m always already up at a certain time and go to bed at 7, and that’s right when I take it”. Another said, “every single night I prep my pills for the next day, and I have them all out of their bottles”.

#### 3.7.2. Reminders

Participants reported that reminders were helpful. One participant described visual cues, stating “I put [a sticky note] on my computer and it stays there, you know, for a while. Now I remember”. Another participant reported that physical cues helped reinforce adherence, as they remembered when “it starts hurting”. Participants’ social support also served as reminders, with one person saying, “Uh, it’s my mom. She always says, ‘Did you take your medication?’”.

#### 3.7.3. Technology

Many participants used adherence-promoting technology. Some used a phone alarm as a reminder for medication (“Setting my alarm, you know, on my phone and just taking it… at a certain—at time”) and eating (“I have it—an alarm set for food”). Others used medication adherence apps, e.g., “My daily reminder on my phone, which is the app called Medisafe”.

#### 3.7.4. Support from Others

Participants described social support as helpful for adherence. One participant shared, “tell family and friends, so they know, ‘hey, [NAME] can’t eat at this time’. Or ‘[NAME] can only do this at this time’. So, they are aware”. Another described their work environment as supportive, saying, “Everybody knows at 5 PM my phone rings, I am out of the meeting. I go take my medication and I come back”.

#### 3.7.5. Distraction

One participant described distracting themselves during food abstinence windows, noting, “I often, like, will plan things around [dose times]. Just so if I take the medicine, I’ll go for a walk, so I have an hour [before I can eat]. It helps the time pass by faster”. Another patient said, “Just trying to plan things around that time. I often have to, you know either, go longer without food than I’d like”.

#### 3.7.6. Planning for Side Effects

Planning for medication side effects helped one participant adhere to the selumetinib. This participant said, “And planning for those side effects. So, whether, like I said, knowing ahead of time that I get nauseous, I’ll take Zofran first”.

#### 3.7.7. Belief the Drug Is Helping

Believing that the study drug was helping was another facilitator of adherence. One participant who had experienced a drug hold said, “because they took me off the drugs for a brief period of time, and I felt, again, what it was like not to be on it, I can definitely say, pain wise, it is definitely worth it to be on the drug”.

#### 3.7.8. Wanting to Help Research

For one patient, being a study participant was a facilitator. The patient said, “I want to be a good, I guess, guinea pig, so I’m trying to do my best”.

#### 3.7.9. Memory/Remembering

One individual described their memory as a facilitator. This person said, “And my memory—I still remember that I need to take it”.

#### 3.7.10. Keeping Medication with Them

Finally, keeping medication with the participants was an identified facilitator. One person commented, “Since with that [MEMS] cap and all, I cannot split it. Like I cannot put one in my pocket or leave some at home, leave some at work, you know. So, I have to carry the bottle with me all the time”. Another described, “I usually did take the bottle with me, or I just excused myself, I get to home so I can take it on time”.

### 3.8. Barriers to Adherence Questionnaire

Consistent with findings from the qualitative interviews, the most endorsed barrier on the Barriers to Adherence Questionnaire was “forgetting” (54.55% of participants). Both “busy with other things” and “changes in daily routine” were endorsed by 36.36% of participants. Less frequent barriers were “being away from home” (27.2%), “too tired or slept through dose time” (27.27%), “ran out of pills” (9.09%), and “didn’t feel like it/needed a break” (9.09%). Wanting to avoid side effects, having too many pills to take, feeling sick, or feeling stressed were not endorsed by any participants. Two participants added a barrier in the “other” category, with one describing a drug hold and one describing a specific instance of forgetting.

## 4. Discussion

This pilot study used mixed methods to examine adherence in adults with NF1-PN, with a primary aim of investigating MEMS^TM^ cap feasibility for adherence assessment in adults on a clinical trial of selumetinib. The predetermined target of obtaining data from ≥75% of participants over ≥2 cycles was exceeded, suggesting that MEMS^TM^ caps are feasible. However, unexpected challenges resulted in missing data, including the cap’s battery expiring, cap malfunction, patients forgetting to return the cap, and failure to replace the cap on the next cycles’ bottle. Additionally, one participant withdrew from the adherence protocol because of their difficulties with the MEMS^TM^ caps. Future research may circumvent some of these challenges by using wireless MEMS^TM^ caps [40], which transmit data remotely and display the last opening to the participant.

Contrary to some prior research [21,22,23,24], our study did not find an obvious pattern of reduced longitudinal adherence over time, although we were underpowered to perform statistical comparisons here. Perhaps patients were encouraged to maintain high adherence due to the decreased PN volume and reduced pain documented in this clinical trial [35]. The trial also may have attracted patients more likely to adhere, or provided increased support for adherence compared to other settings. The qualitative results revealed many adherence facilitators in this sample, including consistency and routine, reminders, technology, and support from others. Although two participants identified no barriers to adherence, others described forgetting, being too busy, and the timing of doses as barriers.

As research increasingly focuses on treating PN with oral medications such as selumetinib [41,42] and mirdametinib [43], understanding patient experiences of barriers and facilitators to adherence is vitally important. Future research and clinical uses of MEK inhibitors targeting PN reduction may consider these patient experiences when discussing adherence strategies with patients. For example, researchers should encourage patients to develop consistent routines around medication, to set reminders, and to elicit social support. Additionally, medication timing was a common barrier; therefore, future randomized controlled trials could investigate the influence of timing on medication efficacy to determine the necessity of strict timing windows.

Higher depression and stress scores were associated with reduced adherence. These results were statistically significant despite our small sample size. Previous studies of other populations have reported similar findings, relating depressive symptoms (e.g., among people with cardiovascular disease [44], asthma [45], and type 2 diabetes [46]) and stress (e.g., among people with hypertension [47] and HIV [48]) to adherence. It is unsurprising that adherence in individuals with NF1 would be related to these factors. Preventative screening and treatment for depression and stress as strategies to increase adherence are worth considering; if interventions targeting these individuals could be started before or concurrently with drug initiation, adherence problems may be circumvented. With that said, adherence in this small sample was consistently high. Future studies with larger samples are needed to confirm these findings and explore factors that predict nonadherence in more depth.

Adherence interventions have been developed for several illnesses and therapies with varying efficacy [49,50]. While adherence-promoting strategies can be used effectively (e.g., health education, reminders), more research is necessary in the NF population [51]. Using patient-centered data sources, such as qualitative interviews, may help tailor interventions to specific patient populations. Future qualitative research also should examine facilitators and barriers for subpopulations of participants who may be at risk of poorer adherence, such as adults with depressive symptoms or high levels of stress.

Mean MEMS^TM^ adherence rates in our sample were lower than the other methods by at least 12 points across all time periods. Future NF1 clinical trials should use MEMS^TM^ caps to understand patterns of long-term adherence, while considering the challenges of battery expiration, cost, and error. Despite these challenges, MEMS^TM^ caps may have use clinically in identifying people whose adherence drops below a pre-specified level.

### Limitations

The current research was part of a pilot study investigating medication adherence in a trial of selumetinib; therefore, the sample size was intentionally (and necessarily) small. This limits generalizability to the larger NF1 population and was a barrier to exploring medical variables associated with adherence. However, pilot studies are necessary steps in the scientific process to evaluate feasibility and to inform future directions in research [52]. Additionally, the characteristics of individuals who participate in clinical trials tend to be different than those in the general population [53]. Further research is needed to explore facilitators and barriers for larger samples of people with NF1 as more oral medications become available for use outside of clinical trials. Future research should recruit larger samples to further explore MEMS^TM^ feasibility, as well as the baseline characteristics that may predispose individuals to low adherence. Additionally, problems with missing MEMS^TM^ data could be minimized through planning more frequent reminders, such as through a smartphone app. Documenting changes in perceived barriers and facilitators over the course of a trial and determining patient characteristics associated with particular barriers and facilitators are also worthwhile aims for future NF1 studies.

## 5. Conclusions

This pilot study is the first investigation using electronic pill caps to assess medication adherence among adults with NF1. MEMS^TM^ caps proved a feasible method, and our results can inform future clinical trials studying oral medications with an NF1 population. Given the preliminary associations found in this study, providers may consider screening and treatment for depression and stress to increase adherence in both research and clinical settings. Additionally, qualitative data suggest that providers should encourage their patients to elicit social support, create a consistent routine, and use reminders to promote adherence. As medication adherence can be challenging, the continued exploration of factors related to adherence and the identification of other intervention targets to increase adherence among NF1 populations are warranted in larger samples. Larger samples will address potential biases inherent in pilot studies with small sample sizes.

## Figures and Tables

**Table 1:** Participant characteristics.

Participant Characteristics	N (%)
Age (Mean (SD))	34.36 (8.91), N = 12
Education (Mean (SD))	14.83 (1.80), N = 12
Sex Assigned at Birth	
Female	5 (41.6%)
Male	7 (58.3%)
Race/Ethnicity	
White	8 (66.7%)
Black	2 (16.7%)
Asian	1 (8.3%)
American Indian/Alaskan Native	1 (8.3%)
Not Hispanic/Latino/a/e	12 (100%)
Marital Status	
Single	10 (83.3%)
Married	2 (16.7%)
Occupational Status	
Unemployed	3 (25%)
Employed, (Full-time/Part-time)	7 (58.3%)/2 (16.7%)
Psychological/Neurological History	
Attention Deficit (ADHD)	2 (16.7%)
Learning Disability	4 (33.3%)
Depression	2 (16.7%)
Anxiety	3 (25%)

**Table 2:** Adherence rates across three assessment methods.

Cycle	Method	Mean	Standard Deviation	95% Confidence Interval
1–4	MEMS^TM^ (n = 11)	83.39	16.71	72.16–94.62
	Diary (n = 12)	97.88	5.02	94.69–101.07
	Pill counts (n = 12)	98.25	3.22	96.20–100.30
5–8	MEMS^TM^ (n = 10)	87.01	14.62	76.55–97.47
	Diary (n = 12)	99.22	1.54	98.24–100.20
	Pill counts (n = 12)	99.50	0.82	98.98–100.02
9–12	MEMS^TM^ (n = 8)	84.77	15.44	71.86–97.68
	Diary (n = 10)	98.84	1.19	97.99–99.69
	Pill counts (n = 10)	98.51	2.14	96.98 -100.04
13–18	MEMS^TM^ (n = 8)	79.68	20.85	62.25–97.11
	Diary (n = 10)	96.14	5.35	92.31–99.97
	Pill counts (n = 10)	97.53	2.99	95.39–99.67

**Table 3:** Relationship between participant characteristics and adherence.

	MEMS^TM^ Cap	Medication Diary	Pill Count
Number of Stressful Life Events at Baseline			
Cycles 1–4	*r* = −0.035, *p* = 0.919	*r* = −0.546, *p* = 0.066	*r* = −0.399, *p* = 0.199
Cycles 5–8	*r* = 0.227, *p* = 0.528	*r* = −0.304, *p* = 0.337	*r* = −0.320, *p* = 0.310
Cycles 9–12	*r* = 0.179, *p* = 0.672	*r* = −0.494, *p* = 0.147	*r* = −0.086, *p* = 0.813
Cycles 13–18	*r* = 0.041, *p* = 0.923	*r* = −0.186, *p* = 0.606	*r* = −0.246, *p* = 0.494
Rating of Overall Stress at Baseline			
Cycles 1–4	*r* = −0.638, *p* = 0.035 *	*r* = −0.472, *p* = 0.121	*r* = −0.617, *p* = 0.033 *
Cycles 5–8	*r* = −0.361, *p* = 0.306	*r* = −0.325, *p* = 0.302	*r* = −0.323, *p* = 0.306
Cycles 9–12	*r* = −0.407, *p* = 0.317	*r* = −0.106, *p* = 0.772	*r* = 0.198, *p* = 0.584
Cycles 13–18	*r* = −0.180, *p* = 0.670	*r* = 0.108, *p* = 0.766	*r* = 0.136, *p* = 0.708
PROMIS Depression at Baseline			
Cycles 1–4	*r* = −0.607, *p* = 0.048 *	*r* = 0.062, *p* = 0.848	*r* = 0.124, *p* = 0.701
Cycles 5–8	*r* = −0.658, *p* = 0.038 *	*r* = −0.489, *p* = 0.107	*r* = −0.563, *p* = 0.057
Cycles 9–12	*r* = −0.708, *p* = 0.050 *	*r* = −0.556, *p* = 0.095	*r* = −0.503, *p* = 0.138
Cycles 13–18	*r* = −0.878, *p* = 0.004 **	*r* = −0.617, *p* = 0.057	*r* = −0.553, *p* = 0.097
PROMIS Cognitive Function at Baseline			
Cycles 1–4	*r* = 0.293, *p* = 0.382	*r* = −0.255, *p* = 0.424	*r* = −0.326, *p* = 0.302
Cycles 5–8	*r* = 0.278, *p* = 0.436	*r* = 0.088, *p* = 0.784	*r* = 0.108, *p* = 0.737
Cycles 9–12	*r* = 0.229, *p* = 0.586	*r* = 0.019, *p* = 0.959	*r* = 0.100, *p* = 0.784
Cycles 13–18	*r* = 0.530, *p* = 0.177	*r* = 0.147, *p* = 0.686	*r* = 0.181, *p* = 0.617
PROMIS Pain Interference at Baseline			
Cycles 1–4	*r* = −0.215, *p* = 0.526	*r* = 0.047, *p* = 0.884	*r* = 0.018, *p* = 0.955
Cycles 5–8	*r* = 0.049, *p* = 0.894	*r* = −0.077, *p* = 0.813	*r* = −0.125, *p* = 0.698
Cycles 9–12	*r* = −0.048, *p* = 0.911	*r* = 0.130, *p* = 0.721	*r* = 0.374, *p* = 0.287
Cycles 13–18	*r* = −0.167, *p* = 0.693	*r* = 0.178, *p* = 0.622	*r* = 0.239, *p* = 0.506

* *p* < 0.05, ** *p* < 0.01.

## Data Availability

Deidentified data are available in the NF Data Portal (https://nf.synapse.org (accessed on 12 January 2025)).
